# BCRP/ABCG2 Inhibition Sensitizes Hepatocellular Carcinoma Cells to Sorafenib

**DOI:** 10.1371/journal.pone.0083627

**Published:** 2013-12-31

**Authors:** Wei-Chien Huang, Yi-Ling Hsieh, Chao-Ming Hung, Pei-Hsuan Chien, Yu-Fong Chien, Lei-Chin Chen, Chih-Yen Tu, Chia-Hung Chen, Sheng-Chieh Hsu, Yueh-Ming Lin, Yun-Ju Chen

**Affiliations:** 1 Center for Molecular Medicine, China Medical University Hospital, Taichung, Taiwan; 2 Division of Pulmonary and Critical Care Medicine, China Medical University Hospital, Taichung, Taiwan; 3 Graduate Institute of Cancer Biology, China Medical University, Taichung, Taiwan; 4 The Ph.D. program for Cancer Biology and Drug Discovery, China Medical University, Taichung, Taiwan; 5 Department of Internal Medicine, China Medical University, Taichung, Taiwan; 6 Department of Respiratory Therapy, China Medical University, Taichung, Taiwan; 7 Graduate Institute of Clinical Medical Science, China Medical University, Taichung, Taiwan; 8 Department of Biotechnology, Asia University, Taichung, Taiwan; 9 Department of Biological Science & Technology, I-Shou University, Kaohsiung, Taiwan; 10 School of Medicine for International Students, I-Shou University, Kaohsiung, Taiwan; 11 Department of Nutrition, I-Shou University, Kaohsiung, Taiwan; 12 Department of General Surgery, E-Da Hospital, Kaohsiung, Taiwan; 13 Department of Medical Research, E-Da Hospital, Kaohsiung, Taiwan; 14 Department of Life Science, National Chung-Hsing University, Taichung, Taiwan; 15 Division of Colorectal Surgery, Department of Surgery, Kaohsiung Chang Gung Memorial Hospital and Chang Gung University College of Medicine, Kaohsiung, Taiwan; China Medical University, Taiwan

## Abstract

The multikinase inhibitor, sorafenib (Nexavar®, BAY43-9006), which inhibits both the Raf/MEK/ERK pathway and several receptor tyrosine kinases (RTKs), has shown significantly therapeutic benefits in advanced hepatocellular carcinoma (HCC). However, not all HCC patients respond to sorafenib well and new therapeutic strategies to optimize the efficacy of sorafenib are urgently required. Overexpression of breast cancer resistance protein (BCRP/ABCG2) mediates the drug-efflux of several tyrosine kinase inhibitors (TKIs) to attenuate their efficacy. This study aimed to investigate the role of BCRP/ABCG2 in the sensitivity of HCC to sorafenib. Our data showed that BCRP/ABCG2 mediated the efflux of sorafenib. Co-treatment with a BCRP/ABCG2 inhibitor greatly augmented the cytotoxicity of sorafenib in HCC cells. Similar results were also achieved by the competitive inhibitor of BCRP/ABCG2, gefitinib, in combination with sorafenib. These results suggest not only that BCRP/ABCG2 is a potential predictor for the sorafenib sensitivity in HCC, but also that blockage of BCRP/ABCG2 may be a potential strategy to increase the response of HCC cells to sorafenib.

## Background

Hepatocellular carcinoma (HCC) is a leading cause of cancer mortality in the world, especially in Asia[1], [2]. Because there is no obvious symptom during the early stage, HCC patients are often diagnosed at the advanced stage, and the advanced HCC is recognized as a difficult-to-treat disease[3], [4], [5], [6]. The multikinase inhibitor, sorafenib (Nexavar®, BAY43-9006) is now the only drug for the standard treatment of advanced HCC[7], [8]. However, HCC patients show different responses to this drug[9], [10], and the underlying mechanism remains unclear.

ATP-binding cassette (ABC) transporters mediate drug efflux to protect cells from xenobiotic- and toxin-induced damages under physiological conditions. Overexpression of ABC transporters is frequently observed in cancer patients who are unresponsive to chemotherapy, and has been proposed to account for the multidrug resistance (MDR) of cancer cells[11], [12]. Inhibition of ABC transporter activity is a potential strategy to overcome the chemoresistance. Three ABC transporters, including P-glycoprotein (P-gp, MDR1, ABCB1), multidrug resistance protein 1 (MRP1, ABCC1), and breast cancer resistance protein (BCRP, MXR, ABCG2), play important roles in most cases of MDR in cancer cells[13], [14]. In the past few years, small molecule tyrosine kinase inhibitors (TKIs) have been suggested to be potential substrates of ABC transporters and combinatory usage of these TKIs as competitive inhibitors is able to reduce ABC transporter-mediated MDR[15], [16], [17], [18]. Among these transporters, BCRP/ABCG2 overexpression was found to confer resistance to gefitinib, the epidermal growth factor receptor (EGFR) TKI, suggesting the association between ABC transporter expression and TKI resistance[19], [20], [21], [22].

BCRP/ABCG2 and MDR1 are two major regulators controlling the brain distribution of anti-cancer drugs. It has been reported that BCRP/ABCG2 plays a significant role in restricting the distribution of sorafenib across the blood-brain barrier (BBB) to the brain[24], [26], [27]. In comparison to MDR1, BCRP/ABCG2 showed higher activity in the transportation of sorafenib *in vitro* [23], [24], [25]. Although BCRP/ABCG2 and MDR1 have been viewed as the two most important determinants for MDR in response to chemotherapy in HCC[28], [29], however, it remains unclear whether BCRP/ABCG2 expression is associated with HCC sensitivity to sorafenib. Therefore, this study aimed to investigate the causal relationship between BCRP/ABCG2 expression and sorafenib sensitivity, and to examine whether BCRP/ABCG2 inhibition is a potential strategy to sensitize HCC cells to sorafenib.

## Methods

### Cell lines and reagents

Hep3B and HepG2 HCC cell lines were maintained in Dulbecco's modified Eagle's medium/F12 medium supplemented with 10% fetal bovine serum (Logan, UT). Huh-7 HCC cells were maintained in Dulbecco's modified Eagle's medium supplemented with 10% fetal bovine serum. Sorafenib was kindly provided by Dr. Chao-Ming Hung (E-Da Hospital, Kaohsiung, TW) and was dissolved in dimethyl sulfoxide (DMSO) as stock concentration at 100 µM. Chrysin was purchased from Sigma-Aldrich (St. Louis, MO). Gefitinib was purchased from LC laboratory. The BCRP/ABCG2 protein level was detected by using an anti-BCRP antibody from Santa Cruz Biotechnology Inc. (Santa Cruz, CA). The anti-phospho-ERK1/2-T202/Y204 antibody (p-ERK1/2) and anti-cleaved PARP antibody from Cell Signaling (Danvers, MA) were used. Turbofect™ siRNA transfection reagent was purchased from Fermentas (Glen Burnie, MD). TransIT-2020 transfection reagent was purchased from Mirus Bio LLC (Madison, WI).

### Cell viability assay

*In vitro* cell viability assays were conducted by using the 3-(4,5-dimethylthiazol-2-yl)-2,5-diphenyltetrazolium bromide (MTT) colorimetric assay, crystal violet staining or bright-field imaging. For the MTT assay, cells (5×10^3^ cells per well) were seeded in 96-well plates overnight. Cells were subjected to pre-treatment with BCRP/ABCG2 inhibitors, followed by sorafenib treatment. Three days later, relative cell amounts were determined by adding 1 µg/ml MTT to each well. Then, the medium was removed after 4-hour incubation. Formazan solubilized in 100 µl DMSO was added to each well, and the absorbance was measured at 570 nm. For the crystal violet staining assay, HCC cells, subjected to the indicated experiments, were re-seeded (1×10^5^ cells per well) in 6-well plates overnight, followed by sorafenib treatment. Approximately one week later, relative cell amounts were determined by crystal violet staining. Briefly, cells were washed with 1X PBS once, followed by fixation and staining with 1% crystal violet dissolved in 30% ethanol for 15–30 minutes at room temperature. Then, cells were washed with tap water to eliminate background interference.

### Drug-efflux assay

Cells were seeded in 6-cm dish and incubated overnight. The next day, cells were treated with 5 µM sorafenib for 1 h. Then, medium was refreshed without sorafenib, followed by recovery. Whole cell lysates were harvested at the indicated time points of recovery and subjected to Western blot analysis. The reversal from sorafenib inhibition during the recovery period was assessed by detecting the level of ERK1/2 activation with an anti-p-ERK1/2 antibody.

### Transfection assay

Transfections of small-interfering RNA (siRNA) and DNA were conducted by using Turbofect™ siRNA transfection reagent and TransIT-2020 transfection reagent, respectively. According to the manufacturer's instruction, cells with 60–70% confluence were transfected with siRNA or DNA, followed by the indicated experiments.

### Construction of expression vector

The *BCRP/ABCG2* gene was obtained from A549 cells by using the forward primer (5′ *Bam*HI-AAAGGATCCATGTCTTCCAGTAATGTCGA 3′) and the reverse primer (5′ CCCGAATTCTTAAGAATATTTTTTAAGAAATAA-*Eco*RI 3′). *BCRP/ABCG2* gene was subsequently cloned into the pCMV-Tag2B expression vector by using the *Bam*HI and *Eco*RI cutting sites. The sequence of the *BCRP/ABCG2* gene was confirmed by sequencing.

### Statistical analysis

The statistical analysis was performed by Student's *t* test. */#, *p*<0.05; **/##, *p*<0.01; ***/###, *p*<0.001 mean as compared to control groups.

## Results

### BCRP/ABCG2 was a determinant for the sensitivity of HCC cells to sorafenib treatment

**Figure 1:**
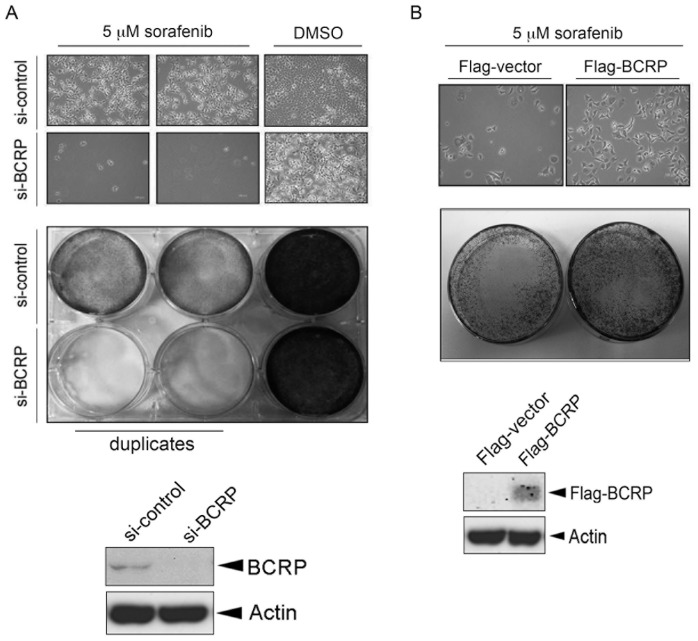
BCRP/ABCG2 is involved in the determination of sorafenib sensitivity in Hep3B hepatocellular carcinoma (HCC) cells. (A–B) Hep3B cells were either knocked down with BCRP siRNA (A) or transfected with Flag-BCRP expression vector (B). One day later, cells were re-seeded at the same density, followed by treatment of 5 µM sorafenib. Three-to five days later, cell viability was measured by using bright-field imaging (*top panel*) and crystal violet staining assay (*middle panel*). BCRP/ABCG2 expression was detected by Western blot analysis (*bottom panel*).

To address whether BCRP/ABCG2 expression is associated with the sensitivity of HCC to sorafenib, BCRP siRNA was employed. Hep3B HCC cells were transfected with control or BCRP siRNA for 24 hours followed by treatment with sorafenib. The effect of sorafenib on cell viability was determined by both bright-field imaging and 1% crystal violet staining. The decreased expression of BCRP/ABCG2 in the cells was confirmed by Western blot analysis, and was found to render Hep3B cells more sensitive to sorafenib (Figure 1A, bright-field image in the *top panel*; crystal violet staining in the *middle panel*). Similar result was also observed in another HepG2 HCC cell line (Figure S1 in File S1). In contrast, sorafenib-induced cytotoxicity was less obvious when BCRP/ABCG2 was overexpressed in Hep3B cells (Figure 1B). Taken together, these results suggest BCRP/ABCG2 as an important determinant for the sensitivity of HCC cells to sorafenib.

### BCRP/ABCG2-mediated sorafenib efflux was observed in HCC cells

**Figure 2:**
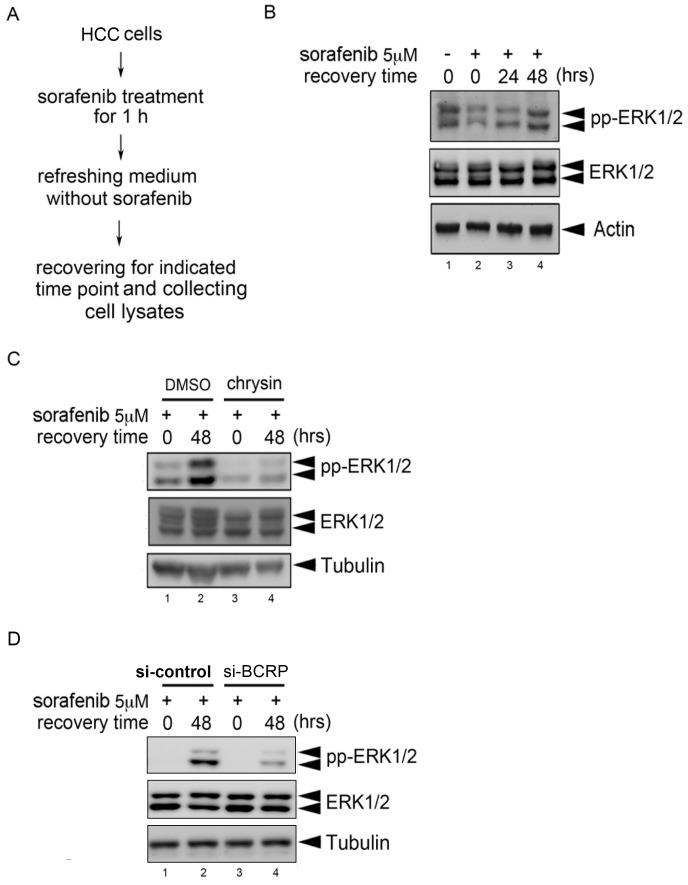
BCRP/ABCG2 mediates the drug efflux of sorafenib in HCC cells. (A) The experimental procedure of the drug-efflux assay was illustrated. (B) Hep3B cells were subjected to drug-efflux assay. The expression levels of phosphorylated ERK1/2, ERK1/2 and Tubulin were examined by Western blot analysis. (C) Hep3B cells were pre-treated with 25 µM chrysin for 1 h, followed by the drug-efflux assay. The expression levels of phosphorylated ERK1/2, ERK1/2 and Tubulin were examined by Western blot analysis. (D) HepG2 cells were transiently transfected with either control siRNA or BCRP siRNA for 4 days, followed by the drug-efflux assay. The expression levels of phosphorylated ERK1/2, ERK1/2 and Tubulin were examined by Western blot analysis.

We further examined whether the drug-efflux effect of BCRP/ABCG2 affects the anti-tumor effect of sorafenib in HCC cells. Therefore, a drug-efflux assay was designed and performed. Briefly, Hep3B cells were treated with sorafenib for 1 hr followed by medium refreshment without sorafenib and further incubation for 24 or 48 hours to recover cells from the inhibition by sorafenib. Whole cell lysates were then collected at the indicated time points (Figure 2A). Because sorafenib is an inhibitor of Raf-MEK1-ERK1/2 pathway, the phosphorylation level of ERK1/2 was used as an indicator of sorafenib activity. As shown in Figure 2B, inhibition of ERK1/2 phosphorylation by sorafenib (*lane 2*) was gradually recovered in a time-dependent manner (*lanes 3*–*4*), suggesting the existence of sorafenib efflux in Hep3B cells. To determine whether this drug-efflux effect was mediated by BCRP/ABCG2, the BCRP/ABCG2 inhibitor, chrysin, was used. The results showed that the recovery of ERK1/2 phosphorylation from inhibition by sorafenib was observed in Hep3B cells treated with vehicle DMSO (Figure 2C, compared *lane 2* with *lane 1*). However, this recovery was not observed when BCRP/ABCG2 activity was blocked by chrysin (Figure 2C, compared *lane 4* with *lane 3*). Furthermore, our data showed that chrysin itself did not directly inhibit basal ERK1/2 phosphorylation in HCC cells (data not shown). Therefore, it excluded the possibility that chrysin prevents the recovery of ERK activity from sorafenib withdrawal is due to the directly inhibitory effect of chrysin on ERK activation. Similar results were also obtained in HepG2 and Huh-7 HCC cell lines (Figures S2A-B in File S1). To strengthen the importance of BCRP/ABCG2 in this regulation, the BCRP siRNA was used. As shown in Figure 2D, the recovery induction of ERK1/2 phosphorylation from inhibition by sorafenib was dramatically attenuated when the BCRP/ABCG2 expression in HepG2 cells was suppressed by BCRP siRNA (compared *lanes 3*–*4* with *lanes 1*–*2*). Consistently, the similar result was also observed in Huh-7 cells (Figure S2C in File S1). Collectively, these results suggest that the anti-cancer activity of sorafenib was attenuated at least in part by BCRP/ABCG2-mediated drug efflux in HCC cells.

### BCRP/ABCG2 inhibitors augmented the anti-cancer activity of sorafenib in HCC cells

**Figure 3:**
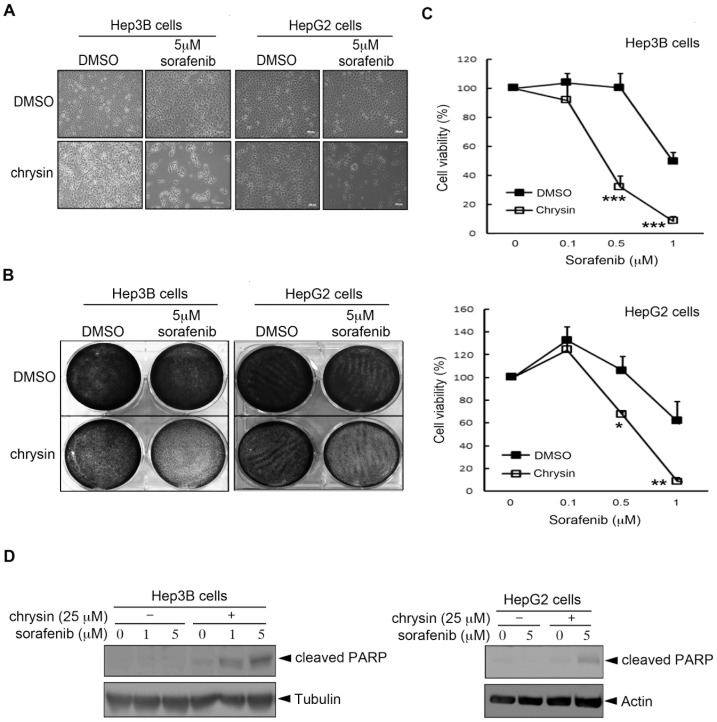
Co-treatment with the BCRP/ABCG2 inhibitor, chrysin, greatly enhances the cytotoxicity of sorafenib in Hep3B and HepG2 HCC cells. (A-D) HCC cells were pre-treated with 25 µM chrysin for 1 h, followed by sorafenib treatment. Cell viability was examined by using a bright-field imaging assay after 1 day (A), crystal violet staining assay after 1 day (B) and MTT assay after 3 days (C). The expression of the apoptotic marker, cleaved PARP, was examined by Western blot analysis (D).

Since our results indicated that BCRP/ABCG2-mediated drug efflux reduced the anti-tumor activity of sorafenib in HCC cells (Figures 1 and 2), we next addressed whether combination with BCRP/ABCG2 inhibitors is a potential strategy to increase the sensitivity of HCC cells to sorafenib. Indeed, our results showed that co-treatment with chrysin synergized the sorafenib-mediated inhibition of cellular viability in both Hep3B and HepG2 HCC cells (Figure 3A). In addition to the bright-field imaging assay, this synergistic effect of chrysin was observed by crystal violet staining (Figure 3B) and MTT assay (Figure 3C). Similar results were also obtained in Huh-7 HCC cells (Figure S3 in File S1). Furthermore, sorafenib only slightly induced the protein cleavage of poly ADP-ribose polymerase (PARP), an apoptotic marker, in Hep3B and HepG2 cells, and this effect was obviously enhanced by co-treatment with chrysin (Figure 3D). Altogether, these results suggest that a combination of BCRP/ABCG2 inhibitor may provide a way to enhance the sensitivity of HCC cells to sorafenib.

### Gefitinib acted as a competitive BCRP/ABCG2 inhibitor to improve the therapeutic efficacy of sorafenib in HCC cells

**Figure 4:**
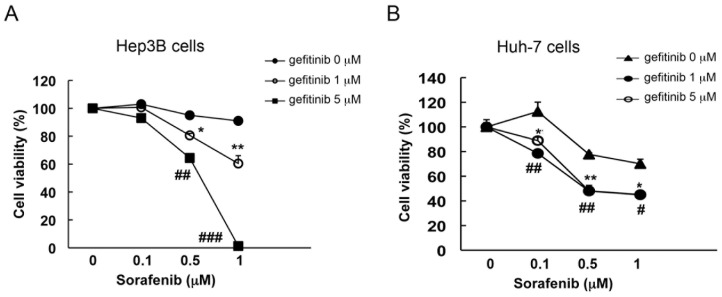
Co-treatment with the BCRP/ABCG2 substrate, gefitinib, enhances the cytotoxicity of sorafenib in Hep3B and Huh-7 HCC cells. (A–B) HCC cells were pre-treated with 1 or 5 µM gefitinib for 1 h, followed by various doses of sorafenib treatment. Three days later, cell viability was examined by MTT assay.

Based on the aforementioned results, simultaneous inhibition of BCRP/ABCG2 activity was suggested to enhance the anti-tumor activity of sorafenib in HCC cells. Due to the binding competition, some substrates for BCRP/ABCG2 have also been recognized as inhibitors of BCRP/ABCG2 when other BCRP/ABCG2 substrates were used simultaneously[30]. Therefore, co-treatment with other anti-cancer drugs, which were also defined as BCRP/ABCG2 substrate, may be an alternative way to enhance the anti-tumor efficacy of sorafenib in HCC cells. EGFR TKI gefitinib was demonstrated as a potential substrate for BCRP/ABCG2[15], [16], [17], [18], and combinatory treatment with gefitinib was also found to overcome the BCRP/ABCG2-mediated resistance to some anti-cancer drugs[31], [32], [33], [34], [35]. Accordingly, we assessed whether gefitinib could be used as an alternative BCRP/ABCG2 inhibitor to increase the cytotoxicity induced by sorafenib in HCC cells. We found that cell viability inhibition by sorafenib was greatly enhanced as the concentration of gefitinib was increased in Hep3B cells (Figure 4A). Consistently, similar result was also observed in both Huh-7 (Figure 4B) and HepG2 cells (Figure S4 in File S1). Taken together, these results suggest that gefitinib may be a promising combinatory therapy for increasing the therapeutic efficacy of sorafenib in HCC.

## Discussion

The approval of sorafenib is a breakthrough for the treatment of HCC. Although it benefits some HCC patients, the most optimized use of sorafenib is not achieved[36]. Thus, many efforts have been made to identify the potential predictors for sorafenib response in order to further elevate its therapeutic efficacy in HCC patients. To date, only a few biomarkers predicting the sensitivity to sorafenib have been identified[37]. Constitutive activation of Akt and signal transducer and activator of transcription 3 (STAT3) have been reported to be associated with sorafenib resistance in HCC[38], [39]. Furthermore, favorable response to sorafenib has been observed in HCC patients with higher level of basal ERK1/2 phosphorylation[40], [41]. More recently, it has been reported that EGFR and HER3 (also known as EGFR3, ErbB3) determine the sensitivity of HCC cells to sorafenib treatment[42], [43]. In the current study, we identified BCRP/ABCG2 as another potential biomarker that might be used to predict the therapeutic efficacy of sorafenib in HCC cells. It has been reported that Akt activity regulates the membrane distribution of BCRP/ABCG2, which may affect its extrusion ability[44]. Our previous studies demonstrated that the induction of BCRP/ABCG2 expression by constitutively activated Akt accounts for the acquired gefitinib resistance[19], [20]. It is worthy to further investigate whether BCRP/ABCG2 expression is also induced in response to chronic treatment with sorafenib and thereby contributes to the Akt-mediated intrinsic and acquired resistance to sorafenib in HCC [39].

In the past three decades, the major focus of researches regarding ABC efflux pumps has been on their roles in mediating chemo-resistance, which is an important challenge for cancer therapy. Many efforts are being made to develop specific and selective inhibitors for ABC transporter, which could not only circumvent the severe MDR to chemotherapy but also have fewer side effects on normal cells. However, none of them have been successfully performed in clinic to date[45]. The successful development of targeted therapy by using small molecule TKIs against critical oncogenes in tumors has brought great improvements to cancer therapy. In addition to chemotherapeutic agents, ABC transporters also efficiently mediate the drug efflux of these TKIs, including gefitinib and sorafenib, and thereby lead to the TKI resistance in tumors[15], [19]. In the current study, we observed that BCRP/ABCG2 mediated the efflux of sorafenib, which in turn, attenuated the response of HCC cells to sorafenib. Co-treatment with a BCRP/ABCG2 inhibitor or substrate greatly augmented the therapeutic efficacy of sorafenib. These results provide the evidence supporting BCRP/ABCG2 as a potential determinant for the sensitivity of HCC to sorafenib. Interestingly, a more recent study suggests that the attenuation of exposure to sorafenib over time in HCC patients may be due to an induction of expression of the efflux transporter in the gut wall. Therefore, an increase in the dose of sorafenib may be considered to elevate its anti-tumor efficacy[46]. Coupling this study with our findings strongly suggests that simultaneous inhibition of BCRP/ABCG2 activity is a potential strategy to augment the sorafenib efficacy in HCC. While BCRP/ABCG2 is demonstrated as a major transporter for the efflux of sorafenib in this and in other studies[24], [26], [27], the possibility of the involvement of other ABC transporters cannot be completely ruled out.

Like the action of chrysin in inhibiting BCRP/ABCG2, our findings showed that the use of gefitinib in combination with sorafenib also enhanced sorafenib-induced cytotoxicity in HCC cells, providing an alternative way to enhance the effectiveness of sorafenib in HCC. Gefitinib is a safe and well-tolerated TKI originally approved for non-small cell lung cancer (NSCLC). More importantly, it has been reported that direct activation of EGFR/HER3 by either autocrine or paracrine signaling circuit is both an adaptive process and a driving force to maintain ERK and Akt activities and subsequent HCC cell growth under sorafenib treatment[42], [43]. As mentioned above, EGFR downstream signaling Akt may regulate both protein expression and membrane distribution of BCRP/ABCG2 to affect its efflux ability[20], [44]. Accordingly, gefitinib may not only function as an EGFR inhibitor to block EGFR/HER3 activation and its downstream survival signaling, but also act as a BCRP/ABCG2 inhibitor by reducing its protein expression and pump activity to prevent the sorafenib efflux from HCC cells. These findings suggest that combination with gefitinib may reduce or prevent the acquisition of sorafenib resistance due to these functions. In supporting to this notion, the results from a phase I clinical trial of sorafenib in combination of gefitinib reveals not only the safety and well tolerance, but also the promising efficacy in recurrent NSCLC patients[47]. Since there is no BCRP/ABCG2 inhibitor approved for clinical use due to the severe side effects on normal cells to date, the combination therapy of gefitinib and sorafenib accordingly seems to be a potential strategy for treatment of advanced HCC.

## Conclusion

Our study shows that BCRP/ABCG2 may be a biomarker for the determination of response to sorafenib in HCC *in vitro*. Simultaneous inhibition of BCRP/ABCG2 activity increases the cytotoxicity of sorafenib in HCC cells. Our findings not only identify a potential predictor for sorafenib sensitivity but also provide a strategy to enhance sorafenib efficacy and decrease the resistance to sorafenib in HCC.

## Supporting Information

File S1 **Supporting Information.** **Figure S1, BCRP/ABCG2 is involved in the determination of sorafenib sensitivity in HepG2 HCC cells.** HepG2 cells were transfected with control siRNA or BCRP siRNA. One day later, cells were re-seeded at the same density, followed by treatment of 5 µM sorafenib. Three days later, cell viability was measured by using crystal violet staining assay (*left panel*). BCRP/ABCG2 expression was detected by Western blot analysis (*right panel*). **Figure S2, BCRP/ABCG2 mediates the drug efflux of sorafenib in HepG2 and Huh-7 cells.** (A–B) HepG2 (A) and Huh-7 (B) cells were pre-treated with 25 µM chrysin for 1 h. Then, the medium was changed to medium lacking sorafenib. Cells were allowed to recover at 0 and 48 hrs time points. The expression levels of phosphorylated ERK1/2, ERK1/2 and Tubulin were examined by Western blot analysis. Fold degree of reversal of sorafenib inhibition on ERK1/2 phosphorylation was shown in *right panel*. (C) Huh-7 cells were transiently transfected with either control siRNA or BCRP siRNA for 4 days, followed by the drug-efflux assay. The expression levels of phosphorylated ERK1/2, ERK1/2 were examined by Western blot analysis. **Figure S3, Co-treatment with the BCRP/ABCG2 inhibitor, chrysin, significantly enhances the cytotoxicity of sorafenib in Huh-7 cells.** (A–B) Huh-7 cells were pre-treated with 25 µM chrysin for 1 h, followed by sorafenib treatment. Cell viability was examined by using crystal violet staining assay after 2 day (A) and MTT assay after 3 days (B). **Figure S4, Co-treatment with the BCRP/ABCG2 substrate, gefitinib, enhances the cytotoxicity of sorafenib in HepG2 cells.** HepG2 cells were pre-treated with 1 or 5 µM gefitinib for 1 h, followed by various doses of sorafenib treatment. Three days later, cell viability was examined by MTT assay.
(DOC)
