# Supplementary material for: BCRP/ABCG2 Inhibition Sensitizes Hepatocellular Carcinoma Cells to Sorafenib
Source: PLoS One. 2013 Dec 31;8(12):e83627. doi: 10.1371/journal.pone.0083627 (PMC3877048; doi:10.1371/journal.pone.0083627)
Supplement: File S1 — Supporting Information. Figure S1, BCRP/ABCG2 is involved in the determination of sorafenib sensitivity in HepG2 HCC cells. HepG2 cells were transfected with control siRNA or BCRP siRNA. One day later, cells were re-seeded at the same density, followed by treatment of 5 µM sorafenib. Three days later, cell viability was measured by using crystal violet staining assay (left panel). BCRP/ABCG2 expression was detected by Western blot analysis (right panel). Figure S2, BCRP/ABCG2 mediates the drug efflux of sorafenib in HepG2 and Huh-7 cells. (A–B) HepG2 (A) and Huh-7 (B) cells were pre-treated with 25 µM chrysin for 1 h. Then, the medium was changed to medium lacking sorafenib. Cells were allowed to recover at 0 and 48 hrs time points. The expression levels of phosphorylated ERK1/2, ERK1/2 and Tubulin were examined by Western blot analysis. Fold degree of reversal of sorafenib inhibition on ERK1/2 phosphorylation was shown in right panel. (C) Huh-7 cells were transiently transfected with either control siRNA or BCRP siRNA for 4 days, followed by the drug-efflux assay. The expression levels of phosphorylated ERK1/2, ERK1/2 were examined by Western blot analysis. Figure S3, Co-treatment with the BCRP/ABCG2 inhibitor, chrysin, significantly enhances the cytotoxicity of sorafenib in Huh-7 cells. (A–B) Huh-7 cells were pre-treated with 25 µM chrysin for 1 h, followed by sorafenib treatment. Cell viability was examined by using crystal violet staining assay after 2 day (A) and MTT assay after 3 days (B). Figure S4, Co-treatment with the BCRP/ABCG2 substrate, gefitinib, enhances the cytotoxicity of sorafenib in HepG2 cells. HepG2 cells were pre-treated with 1 or 5 µM gefitinib for 1 h, followed by various doses of sorafenib treatment. Three days later, cell viability was examined by MTT assay. (DOC) [file pone.0083627.s001.doc]

**File S1: Supporting Information**

**
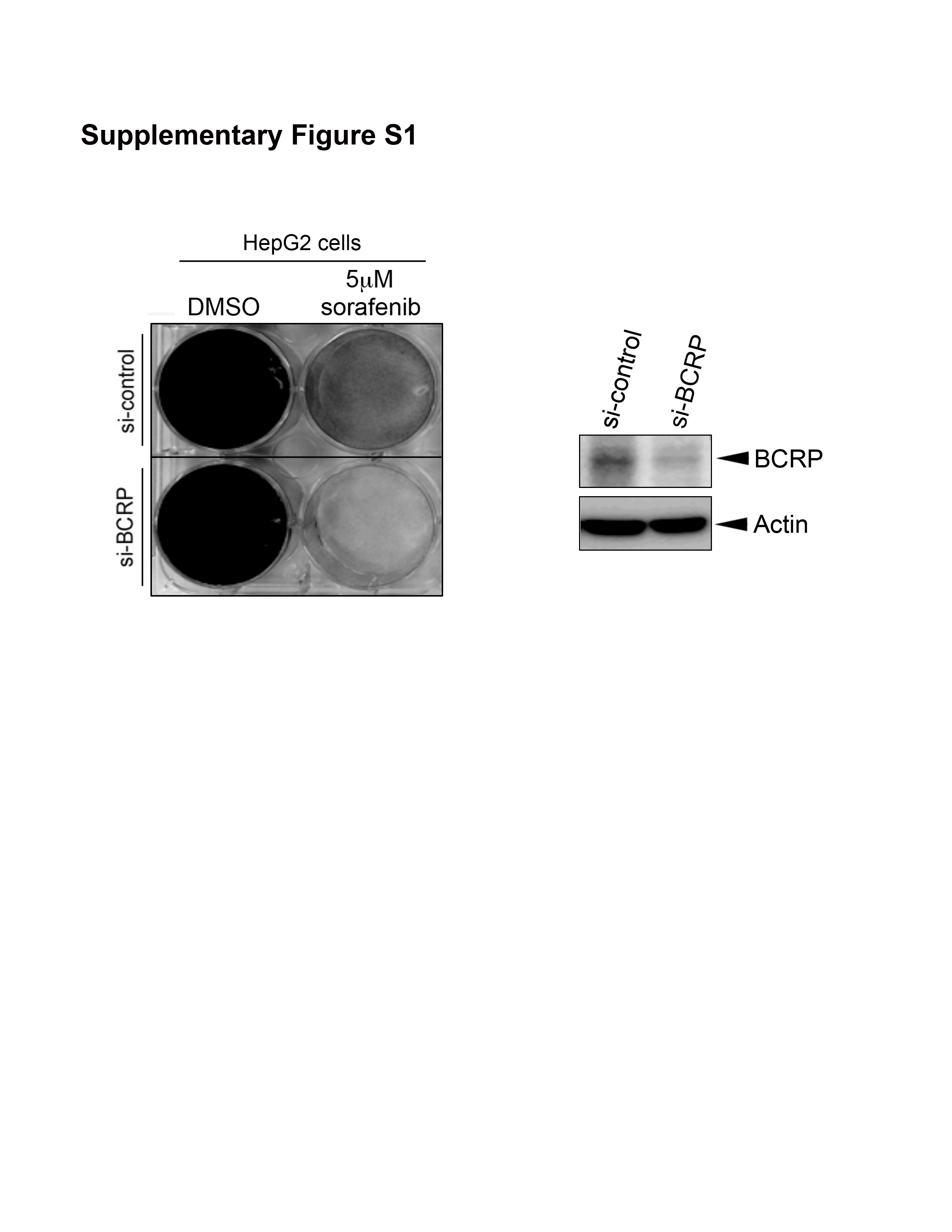
**

**Figure S1. BCRP/ABCG2 is involved in the determination of sorafenib sensitivity in HepG2 HCC cells.** HepG2 cells were transfected with control siRNA or BCRP siRNA. One day later, cells were re-seeded at the same density, followed by treatment of 5 μM sorafenib. Three days later, cell viability was measured by using crystal violet staining assay (*left panel*). BCRP/ABCG2 expression was detected by Western blot analysis (*right panel*).

**File S1: Supporting Information**


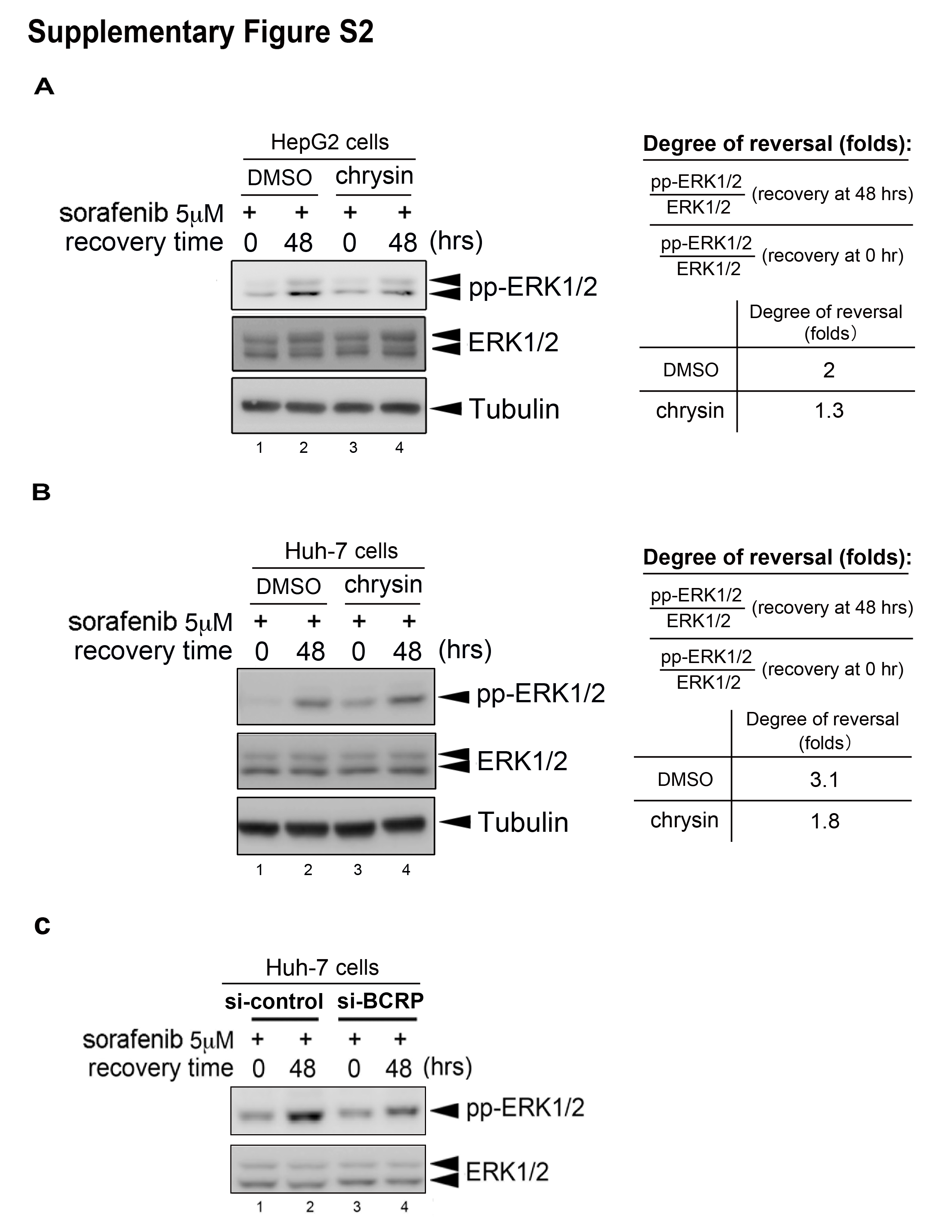


**Figure S2. BCRP/ABCG2 mediates the drug efflux of sorafenib in HepG2 and Huh-7 cells.** (A-B) HepG2 (A) and Huh-7 (B) cells were pre-treated with 25 μM chrysin for 1 h. Then, the medium was changed to medium lacking sorafenib. Cells were allowed to recover at 0 and 48 hrs time points. The expression levels of phosphorylated ERK1/2, ERK1/2 and Tubulin were examined by Western blot analysis. Fold degree of reversal of sorafenib inhibition on ERK1/2 phosphorylation was shown in *right panel*. (C) Huh-7 cells were transiently transfected with either control siRNA or BCRP siRNA for 4 days, followed by the drug-efflux assay. The expression levels of phosphorylated ERK1/2, ERK1/2 were examined by Western blot analysis.

**File S1: Supporting Information**

**
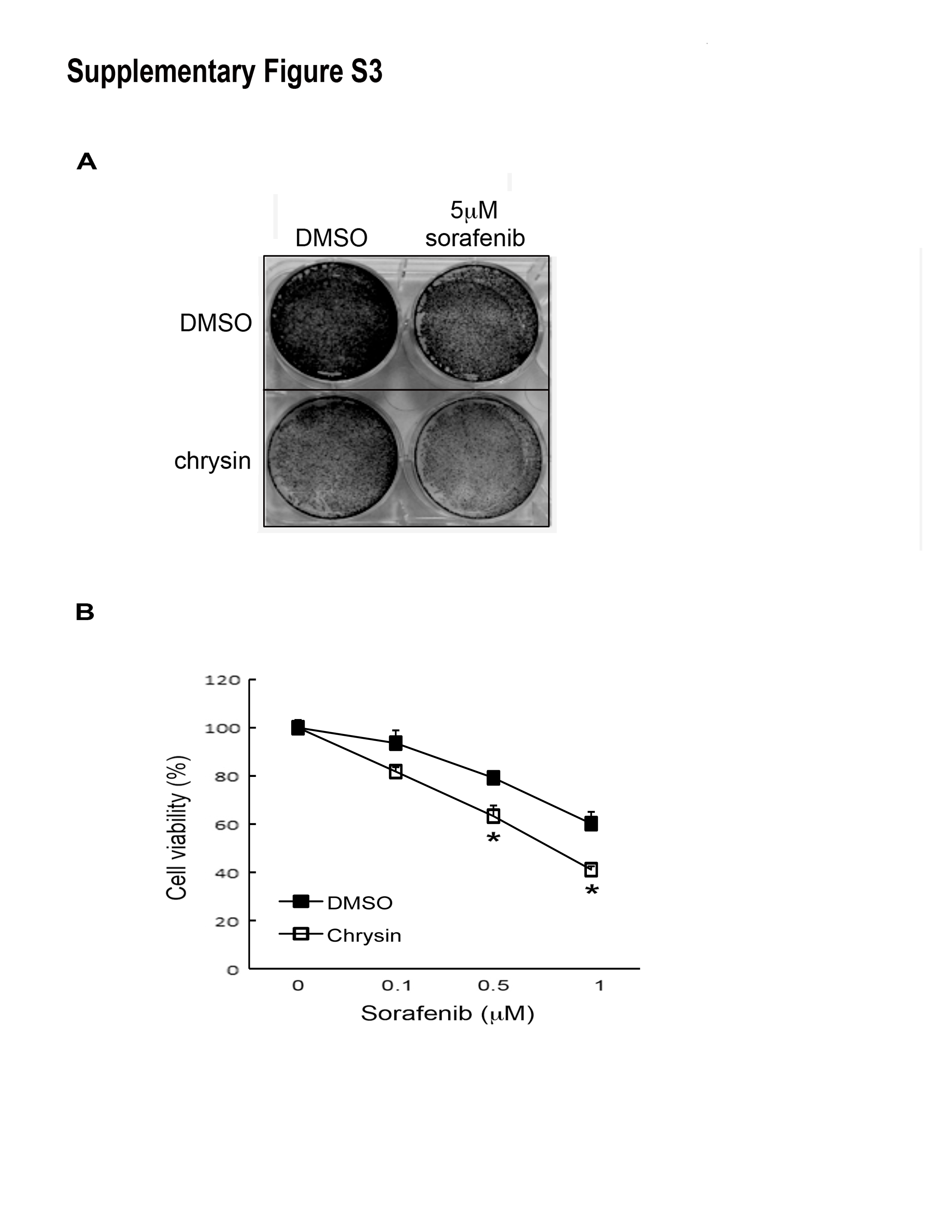
**

**Figure S3. Co-treatment with the BCRP/ABCG2 inhibitor, chrysin, significantly enhances the cytotoxicity of sorafenib in Huh-7 cells.** (A-B) Huh-7 cells were pre-treated with 25 μM chrysin for 1 h, followed by sorafenib treatment. Cell viability was examined by using crystal violet staining assay after 2 day (A) and MTT assay after 3 days (B).

**File S1: Supporting Information**

**
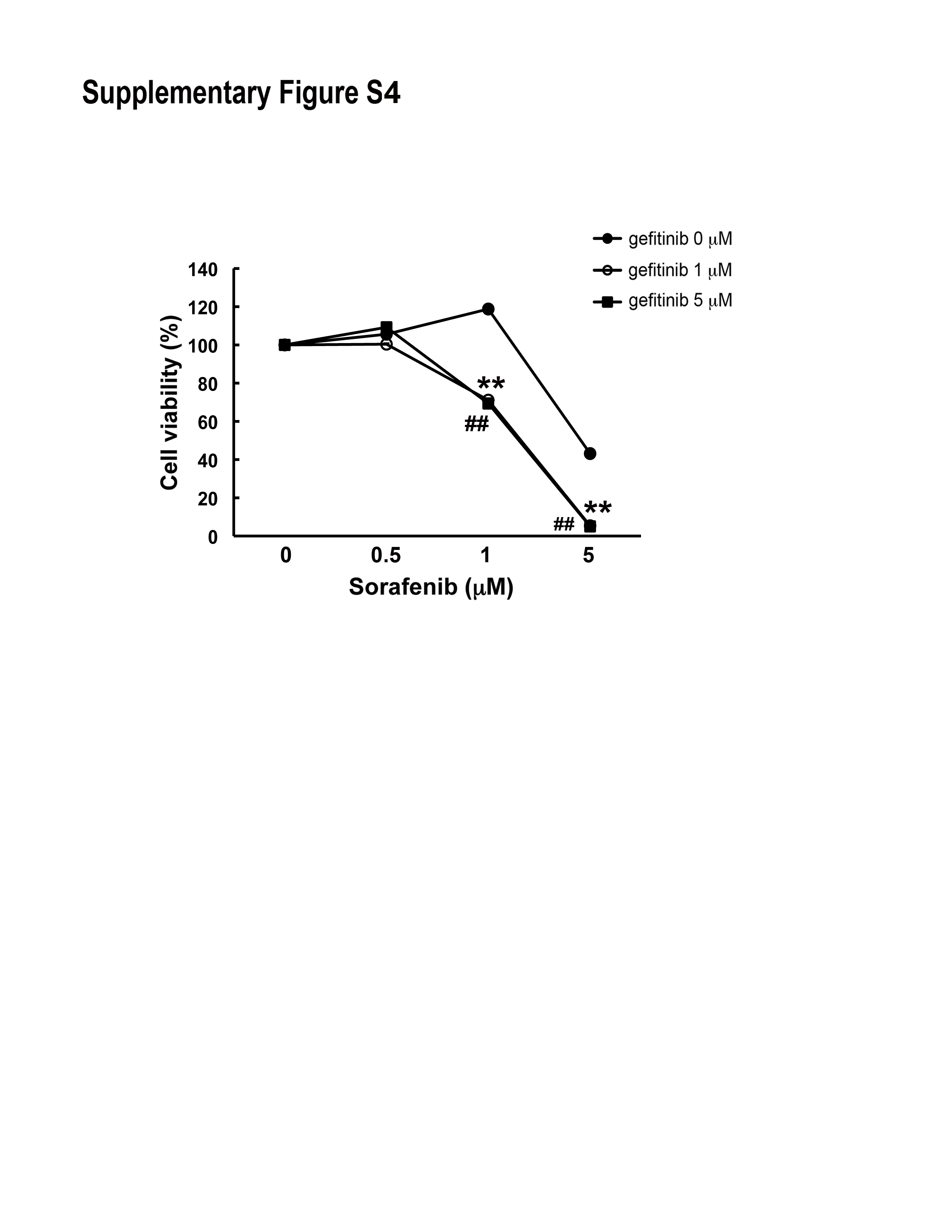
**

**Figure S4. Co-treatment with the BCRP/ABCG2 substrate, gefitinib, enhances the cytotoxicity of sorafenib in HepG2 cells.** HepG2 cells were pre-treated with 1 or 5 μM gefitinib for 1 h, followed by various doses of sorafenib treatment. Three days later, cell viability was examined by MTT assay.
